# Affinity binding of chicken apolipoprotein A1 to a novel flax orbitide (linusorb)

**DOI:** 10.1039/c8ra01757c

**Published:** 2018-05-15

**Authors:** Pramodkumar D. Jadhav, Youn Young Shim, Martin J. T. Reaney

**Affiliations:** Department of Plant Sciences, University of Saskatchewan Saskatoon SK S7N 5A8 Canada younyoung.shim@usask.ca martin.reaney@usask.ca +1 306 966 5015; Guangdong Saskatchewan Oilseed Joint Laboratory, Department of Food Science and Engineering, Jinan University Guangzhou Guangdong 510632 China; Prairie Tide Chemicals Inc. Saskatoon SK S7J 0R1 Canada

## Abstract

Bioactive orbitides (linusorbs, LOs) from flaxseed (*Linum usitatissimum* L.) were ligated through methionine with resin to form an affinity column. The affinity resin was characterized using elemental analysis and the resin bound 70% of its weight in LOs. Chicken serum was passed over the column and washed to remove non-binding materials. The column was eluted with unbound orbitide to competitively release bound protein. A single 28 kDa protein was found in the affinity binding pool. The protein MW and sequence were identical to apolipoprotein A1 (Apo A1), a major serum protein. Its role includes reverse cholesterol transport and cholesterol efflux. The affinity technique allowed convenient and rapid isolation of Apo A1 with a recyclable affinity column. LO binding to a cholesterol carrier molecule might also help us to understand the mechanism of action of LOs in health and the biological activity of flaxseed products.

## Abstract 2

Bioactive orbitides (linusorbs) from flaxseed (*Linum usitatissimum* L.) were ligated through methionine with resin to form an affinity column that selectively binds chicken apolipoprotein A1 from chicken serum.

## Graphical abstract

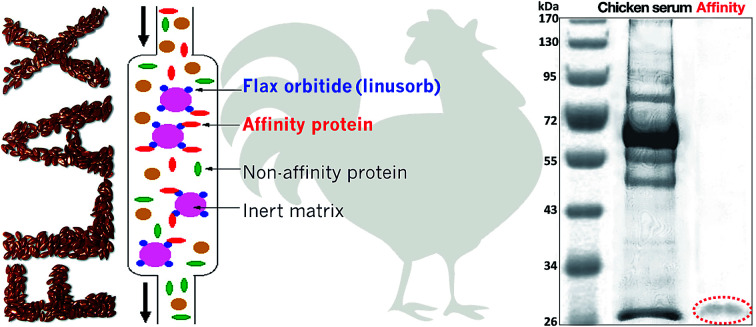

## 1. Introduction

**Fig. 1:**
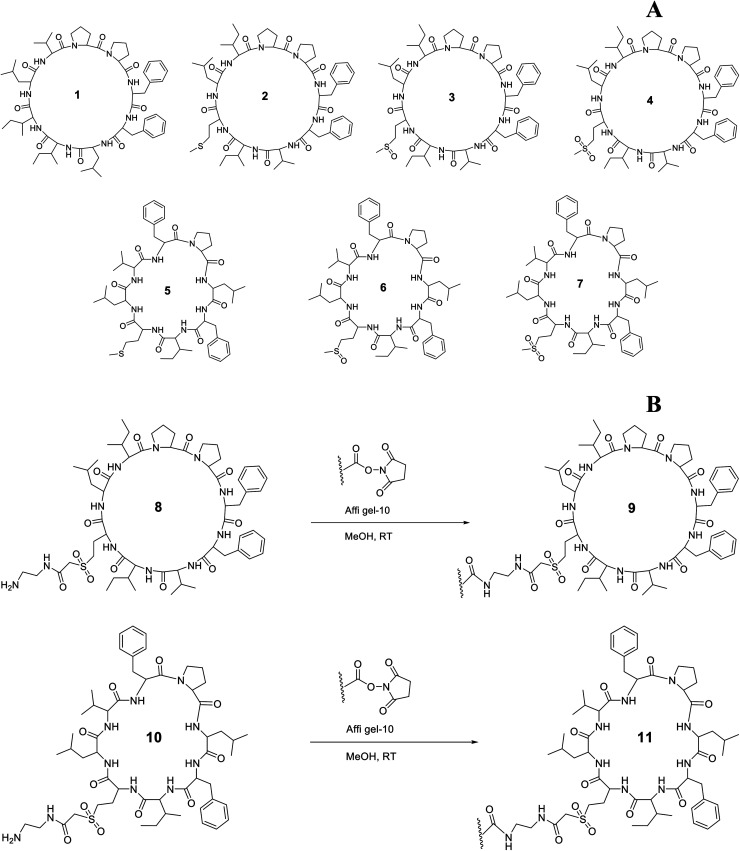
Chemical structure of LOs 1–7 (A); coupling of 8 and 10 with Affi-Gel 10 (B).

A wide range of biological activity has been ascribed to flaxseed (*Linum usitatissimum* L.) products and strong links have been developed that associate flaxseed compounds to their biological activity. In spite of clear evidence of positive health effects of flaxseed, flaxseed meal, flaxseed lignan and flaxseed oil, virtually all studies have failed to control the presence of orbitides, minor biologically active substances. Linusorbs (LOs), orbitides specific to flaxseed, are present in flaxseed oil and are known to suppress immunity and exhibit cytotoxicity against various cancer cell lines.^1–3^ To date, 28 LOs having molecular weights of approximately 1 kDa have been isolated from flaxseed, flaxseed meal, and flaxseed oil. LOs discovered thus far have eight to ten amino acids.^4–9^ The selected structures of LOs 1–7 were shown in Fig. 1A. LOs (2, 3 and 4) and (5, 6 and 7) were analogs of each other with different methionine oxidation state. 1 has shown binding with human serum albumin and the analog of 1 has also shown affinity with hepatocellular peptide binding proteins from plasma membrane and cytosol of rats.^10^

1 has shown immunosuppressive activity comparable to cyclosporine A (CsA) and, therefore, it might be used in a clinical setting.^3^ Of all the LOs, 1 suppressed human peripheral blood lymphocyte proliferation after concanavalin A treatment with an IC_50_ of 2.5 μg mL^−1^. Other LO molecules, 4 (25.2 μg mL^−1^) and 7 (28.1 μg mL^−1^) also had some activity in this assay.^11^1 and CsA form complexes with cyclophilin, and thus interfere with the calcineurin system by inhibiting its phosphatase activity.^12^ CsA has been used in organ transplantation, but it has adverse effects including induction of hyperlipidemia and high total cholesterol levels accompanied with low high density lipoprotein (HDL), and apolipoprotein A1 (Apo A1) levels.^13^ CsA also induces dyslipoproteinemia in a mouse model. The condition is typified by elevated plasma cholesterol, triglycerides and Apo B as compare to control through activation of sterol regulatory element-binding protein (SREBP)-2.^14^ It is also an inhibitor of ABCA1 mediated lipid efflux and inhibition is not specific to either cell type or species. It inversely affects the Apo A1 uptake, degradation and resecretion.^15^ It also inhibits the Apo A1 gene expression through the calcineurin pathway.^16^ Apo A1 is a major protein present in the plasma HDL. The main function of Apo A1 is to reverse cholesterol transport and cholesterol efflux. It transports the cholesterol from peripheral tissues to liver, where it is converted to bile salts and excreted.^17,18^ The synthesis of Apo A1 in chicken occurs in liver, kidney, intestine, muscle, brain, skin, tissues and in mammals, it occurs in liver and intestine.^19^ Apo A1 is present in monomeric form in lipid free state in chicken,^20^ but in mammals itself associates in the absence of lipid.^21,22^ Both human and chicken Apo A1 shows functional similarities based on their secondary and tertiary structure.^23^ Chicken Apo A1 has shown similarity with the human Apo A1 with 48% sequence identity and 62% sequence similarity.^24^ It has been shown that an amphiphilic α-helical structure is responsible for lipid binding and mobilization of cholesterol.^23^ Apo A1 transports cholesterol by binding with ATP-binding membrane cassette transport protein A1 (ABCA1).^25^

Apo A1 has been purified using various techniques such as ultracentrifugation chromatography, immunoaffinity or electrophoretic mobility.^26–30^ However, antibodies and other protein ligands offer high specificity and excellent purity, but have some limitations in larger scale isolation.^31^ Peptides belong to important class of affinity tags. Solid matrix can be easily coupled with peptides containing functional groups and regenerated. They have advantage over other tags like antibodies and proteins in terms of cost and stability. Peptides can be readily synthesized from amino acids or isolated from plants. In some cases they can be incorporated in hydrophobic chromatography media in which hydrophobic amino acid residues play an important role. Activated solid matrix can be coupled with peptides containing compatible functional groups and regenerated. If there is leakage during elution, peptides can be easily removed from proteins. However, the challenge is to obtain specific peptide for protein binding. But by screening ligands from phage display peptide library, specific peptides can be obtained.^32–34^ The hydrophobic surfaces and cyclic conformational structure of LOs makes them good candidates to bind proteins. Hydrophobicity of LOs plays a significant role in transport across cell membranes and distribution in tissues and organs. Hydrophobic peptides such as the somatostatin analog 008, linear hydrophobic peptide EMD 55068 and 1 analog, cyclodecapeptide c(-Ala-Lys-Pro-Phe-Phe-Ala-Lys-Pro-Phe-Phe-), have shown binding to proteins in the plasma membrane and cytosol of rat hepatocytes.^10^

To date LOs protein affinity is largely unexplored. Here we describe the preparation of an LO affinity matrix to study serum protein LO interactions. Chicken serum is analogous to other vertebrate sera and our understanding of molecular interactions in this fluid might aid in elucidating the potential effects of LO binding on biological activity. An affinity column was prepared from 4 and, using a competitive binding strategy, protein fractions with affinity were isolated. A single binding protein was purified using electrophoresis and characterized by mass spectrometry (MS).

## 2. Experimental

### 2.1. Chemicals and reagents

Affi-Gel 10 (*N*-hydroxysuccinimide ester of cross-linked succinyl aminoalkyl agarose, ≥10 μmol mL^−1^), iodoacetamide (IAA), and dithiothreitol (DTT) were purchased from Bio-Rad Laboratories (Hercules, CA, USA). Ammonium bicarbonate and HPLC grade acetonitrile were purchased from Fisher Scientific (Fair Lawn, NJ, USA). Coomassie Brilliant Blue G-250 and sinapinic acid were purchased from Sigma Chemical Co. (St. Louis, MO, USA). Enzymatic digestion was performed using sequencing grade trypsin gold (Promega, Madison, WI, USA). All chemicals were of analytical grade. A Milli-Q system (Millipore, Bedford, MA, USA) was used to prepare deionized water for all mobile phases. Amicon Ultra-4 centrifugal filters having 3 K-regenerated cellulose membrane were obtained from Millipore (Carrigtwohill, Ireland).

### 2.2. Serum sample

Chickens were fed on un-medicated basal diet formulated to fulfill nutritional requirements. Blood samples were obtained from the wing vein of 10 weeks old male commercial broiler chickens (Aviagen). Blood samples were allowed to clot for 2 h at room temperature. The clotted material was removed by centrifugation at 1700 × *g* for 10 min. The supernatant sera were collected for analysis. The experimental protocols were approved by the University of Saskatchewan Animal Care Committee. Procedures were performed as per the requirements of the Guide to the Care and Use of Experimental Animals by the Canadian Council on Animal Care.^35^

### 2.3. Instrumentation

A quadrupole time-of-flight (Q-TOF) Global Ultima mass spectrometer (Micromass, Manchester, UK) equipped with a nano-electrospray (ESI) source and interfaced with a nanoACQUITY UPLC solvent delivery system (Waters, Milford, MA, USA) was used for analysis of proteins after digestion with trypsin. Typical Q-TOF parameter settings consist of a capillary voltage of 3850 V, a cone voltage of 120 V, and a source temperature of 80 °C. Further, MicroTOF-Q II Mass Spectrometer (Bruker Daltonik GmbH, Bremen, Germany) equipped with APCI source was used for MS/MS of 8 and 10 (parameters – dry temp-200 °C; vaporizer temp-450 °C; dry gas-8 L min^−1^; nebulizer -1.6 bar; capillary voltage-4000 V).

### 2.4. Preparation of affinity matrix with bound orbitide

LOs containing an amine side chain, 8 and 10, was synthesized from 2 and 5 as described in our previous work.^36^9 and 11 were prepared using methods modified from a published procedure.^37^8 (0.16 g, 0.14 mmol) was dissolved in MeOH (5 mL) and triethylamine (0.5 mL) was added to the solution. A slurry of Affi-gel (8.30 mL settled resin volume, 0.125 mmol, was prewashed with cold iPrOH and MeOH sequentially) made up to 20 mL was added to the peptide solution and agitated on a shaker at 4 °C for 16 h (Fig. 1B). The gel was filtered and washed with cold MeOH and cold water sequentially, and combined filtrates were concentrated. The gel was added to a solution of ethanolamine (6.25 mL, 0.1 M solution in water, 0.625 mmol) in cold water (25 mL) and agitated on a shaker at 4 °C for 4 h. The gel was then filtered and washed with cold water (6 column volumes) and cold 0.2% aqueous NaN_3_ (3 column volumes) and stored at 0 °C in a solution of 0.2% aqueous NaN_3_ to form coupled gel 9. Similarly, 11 was synthesized from 10 using the above procedure. 10 (0.16 g, 0.15 mmol) was dissolved in MeOH (5 mL) and triethylamine (0.5 mL) was added to the solution in the first step of this synthesis.

### 2.5. Characterization (coupling efficiency) of bound ligand and matrix

The coupling efficiency of stationary phase containing coupled modified LOs and matrix can be determined by performing elemental analysis (C, N and S) on the blank gel and the LO-coupled matrix 9 and 11 (Table 1). In this analysis, samples were first fully combusted and combustion gases, such as carbon dioxide, water, nitric oxide and sulfur dioxide, were analyzed. The difference in S% between blank and modified gel was used to determine coupling efficiency. The analysis shows almost 70% and 38% coupling of LOs to the gel in 9 and 11, respectively.

**Table 1:** Elemental analysis of blank and LO gels (9 and 11)

LO	C	N	S
Blank gel	43.10	4.692	0.000
9	46.42	4.201	0.581
11	44.68	3.879	0.293

### 2.6. Affinity purification

Affi-gel coupled peptide (9) was slurry packed into a empty preparative column [POROS PI 20 μm column (4.6 × 100 mm, 1.7 mL), Applied Biosystems]. The packed column was connected to an Agilent 1200 series HPLC system equipped with a quaternary pump, autosampler, degasser and diode array detector (Agilent G1315C/D, 1024-element photodiode array, wavelength range 190–300 nm). Eluting compounds were detected at 280 nm with a 10 nm bandwidth and against a reference signal at 360 nm. The column was first incubated with serum for 3 min and then eluted for 12 min with a buffer of 0.02 M Tris–HCl pH 7.5 (solvent A) to remove unbound protein and finally, eluted with (1 mg mL^−1^ of 4 in 35% MeOH/0.02 M Tris–HCl, pH 7.5, solvent B) to remove bound protein for 15 min and the column is again equilibrated for 10 min with solvent A at a flow rate of 0.5 mL min^−1^. Fractions were concentrated by ultrafiltration through a 3 K-regenerated cellulose membrane in an Amicon Ultra-4 centrifugal filter assembly and finally, the concentrated sample was centrifuged at 10 000 × *g* for 3 min and clear retentate supernatant solution was used for sodium dodecyl sulfate-polyacrylamide gel electrophoresis (SDS-PAGE).

Proteins were separated by SDS-PAGE according to a previous method^38^ using a Mini protean II system (Bio-Rad, Richmond, CA, USA). The concentrations of protein loaded onto each polyacrylamide gel lane were determined using the Bio-Rad protein assay dye reagent (Richmond, CA, USA). A standard curve was prepared with dilutions of 0.1 mg mL^−1^ BSA solution. Protein (10 μg) was applied to each gel electrophoresis lane. An 8% stacking gel buffer (5.28 mL sterile water; 2.03 mL 40% acrylamide; 2.5 mL 0.5 M Tris–HCl pH 6.8; 100 μL 10% SDS; 50 μL 10% APS; 8 μL TEMED) and 5% resolving gel buffer (3.01 mL sterile water; 0.64 mL 40% acrylamide; 1.25 mL 1.5 M Tris–HCl pH 8.8; 50 μL 10% SDS; 100 μL 10% APS; 8 μL TEMED) were used. Prior to loading, samples were diluted with buffer [0.6 mL pH 6.8; 1 M Tris–HCl; 2 mL 10% (w/v), SDS; 5 mL 50% (w/v) glycerol; 0.8 mL, bromophenol blue], and boiled at 95 °C for 5 min. Prepared samples were applied to the polyacrylamide gel. Electrophoresis was conducted for 75 min by application of a constant voltage of 100 V. Subsequently gels were stained with Coomassie brilliant blue for 1 h then destained using 10% acetic acid in MeOH : H_2_O (40 : 60). Gel images were scanned using a flatbed scanner.

### 2.7. LC-ESI MS spectra collection and data analysis of trypsin digest

Protein in-gel digestion is carried out using the MassPrep II Proteomics Workstation (Micromass, UK) following a previously described procedure.^39^ For LC-ESI-MS analyses, the mobile phase was composed of a binary solvent system of A, 0.2% aqueous formic acid and 3% acetonitrile, and B, 95% acetonitrile with 0.2% formic acid. Peptides were desalted with an in-line solid-phase trap column (180 μm × 20 mm) packed with 5 μm resin (Symmetry C18, Waters) and separated on a capillary column (100 μm × 100 mm, Waters) packed with BEH130 C18 resin (1.7 μm, Waters) with the column temperature maintained at 35 °C. To desalt the sample an injection volume of 2 μL was loaded onto the trapping column and a flow rate of 15 μL min^−1^ was applied for 3 min under initial conditions, using A : B 99 : 1 and diverting the flow to waste. After desalting, flow was diverted from the trap column to the analytical column with a linear gradient of 1–10% solvent B at 400 nL min^−1^ for 16 min, followed by a linear gradient of 10–45% solvent B delivered with a flow rate of 400 nL min^−1^ over 30 min. A fast gradient of 45–80% solvent B in 6 min with flow rate of 800 nL min^−1^ was used to clean the column for subsequent injections followed by equilibrating to initial conditions. Samples were analyzed using Data Dependent Acquisition (DDA), which consists of the detection of multiply charged positive ions (*z* = 2, 3, and 4 were used herein) from an MS survey scan. The mass scan range was set from *m*/*z* 400 to 1900 with a scan time of 1 second.

Data were processed using ProteinLynx Global Server 2.4 software (Waters) using RAW files from LC-ESI-MS and LC-ESI-MS/MS. Peak lists were exported in the micromass (.pkl) format, and subsequently submitted to Mascot (Matrix Science Ltd., London, UK) for peptide search against the NCBInr database hosted by National Research Council of Canada (NRC, Ottawa, ON, Canada).

## 3. Results and discussion

**Fig. 2:**
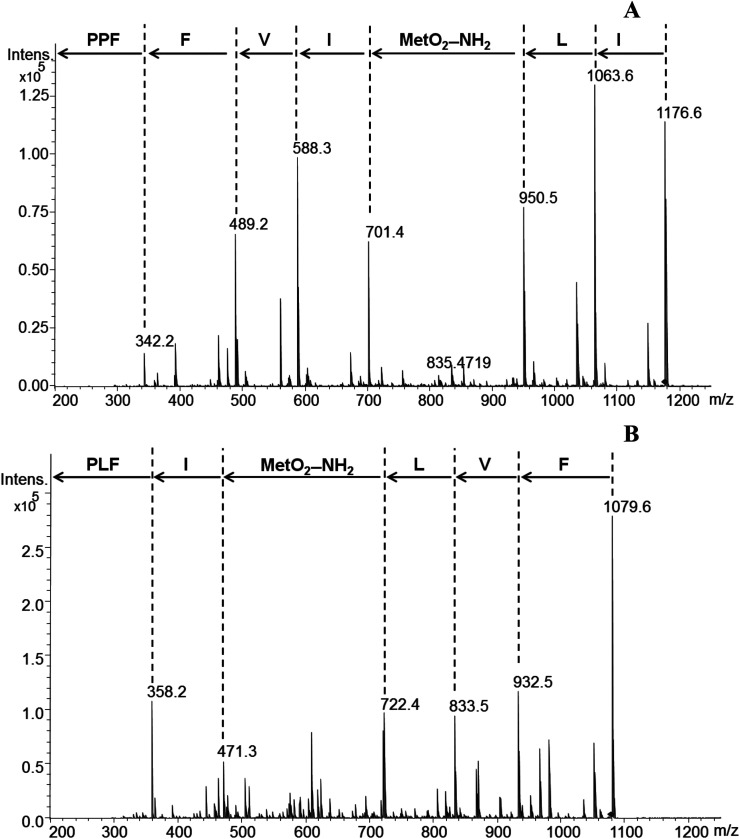
MS/MS fragmentation pattern of 8 (A) and 10 (B). Abbreviations used: P, proline; F, phenylalanine; V, valine; I, isoleucine; MetO_2_–NH_2_, methionine *S*,*S*-dioxide amine; L, leucine.

LOs and their derivatives are a class of natural products with potential applications in the pharmaceutical industry. Due to their known biological activity they might be useful as drugs, drug leads or drug delivery agents.^1–3^ Flax LOs may resist hydrolysis by proteases and other hydrolytic enzymes, a characteristic that can be exploited in their application as drug delivery agents. Hydrophobicity of LOs plays a significant role in transport across cell membranes in addition to distribution in tissues and organs.^10^1 has shown binding to proteins in the plasma membrane and cytosol of rat hepatocytes. 1 and its analogues have shown two surfaces, the aromatic side chains form a hydrophobic surface while the peptide backbone forms a hydrophilic surface. These surfaces and the cyclic conformational structure helps these compounds to bind proteins.^10^ There was no study on the binding of chicken serum proteins with LO analogs. We therefore synthesized 8 and 10 containing amine group and MS/MS fragmentation pattern is shown in Fig. 2.

### 3.1. Structural changes

**Fig. 3:**
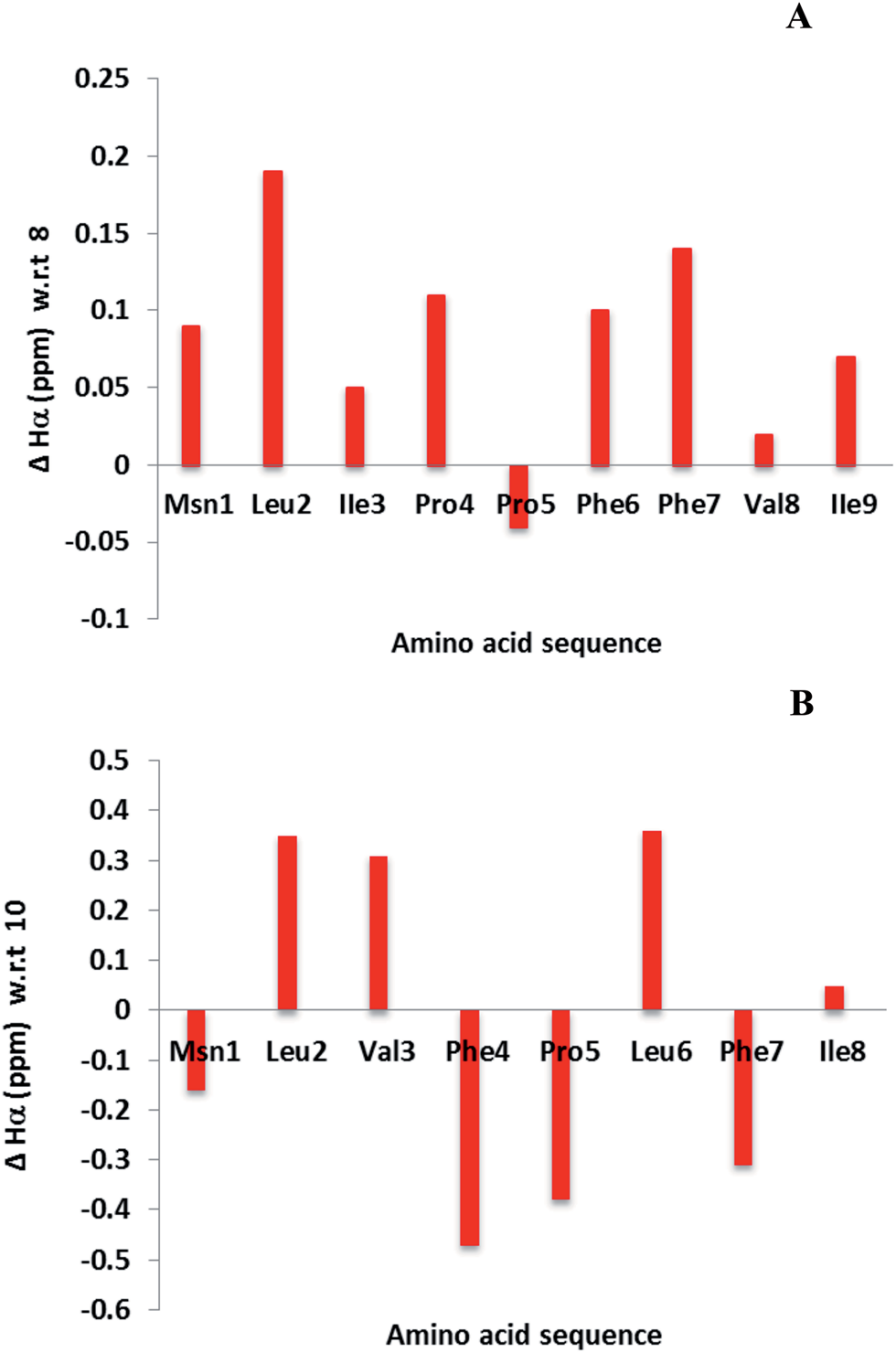
Chemical shift differences of αH signals for LOs 8 (A) and 10 (B) compared with 4 and 7, respectively.

Cyclic peptides have a unique structure that enables them to bind selectively with protein. Hence it is necessary to determine the structural changes between the parent (4 and 7) and modified LOs (8 and 10). The chemical shift differences of αH signals (less than 0.1 ppm) of amino acids in the cyclic peptide indicate both subtle and substantial changes in peptide backbone conformation.^40^ This information is not an alternative to three-dimensional nuclear magnetic resonance (NMR) spectroscopy or X-ray crystallography but can provide complimentary information.^24,40,41^ Some of the examples include α-conotoxin ImI and kalata B1 for detection of structural changes.^42,43^ The difference in αH signals shows that most of the amino acid residues were unperturbed in 8 as compare to 4. In contrast, major changes were observed for 10 as compare to 7 (Fig. 3). It was reported that 10 was present as a single isomer and 7 as two isomers.^36,44^ This may be due to the hydrogen bonding of amino side chain that lock 10 into a single conformation. However, coupling with the affinity resin will reduce hydrogen bonding and might present a similar conformation to that of the parent LO.

### 3.2. Affinity column

**Fig. 4:**
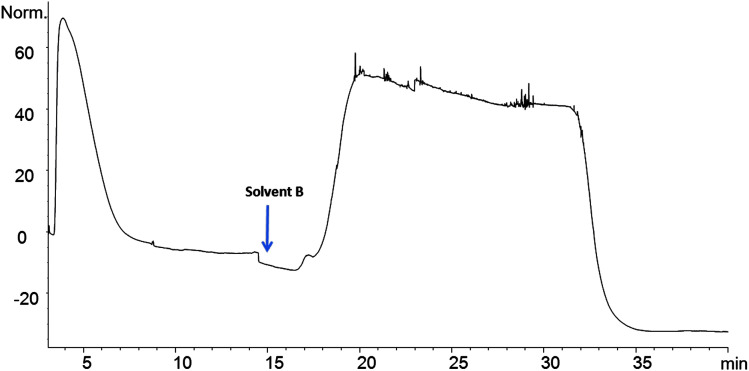
Elution profile of crude serum.

The Affi-Gel 10 matrix was coupled with 8 and 10 through their amine groups. A stable amide bond between LO and matrix occurs with a loss of *N*-hydroxy succinimide. A high coupling efficiency was observed between LO 9 and the matrix and this coupled product was selected for binding studies. The affinity column was connected to an HPLC and solvents were used to sequentially elute the column as described. Eluting materials were monitored by observing absorbance of UV light (280 nm). Chicken serum (2 μL) was passed through the LO 9 matrix column. The chromatogram shows two broad absorption peaks. The first eluting peak was largely unbound protein while the latter peak indicates protein that bound (Fig. 4). Initially the column was eluted with 0.02 M Tris–HCl buffer, pH 7.5 to remove unbound protein at a flow rate of 0.5 mL min^−1^. The competitive elution strategy was performed using unbound peptide (4) to remove bound protein. Subsequently the column was eluted with a solution of 4 in Tris buffer to remove bound protein. Elution was performed at different concentration gradients (0.24 mg mL^−1^ to 1 mg mL^−1^) and protein elution occurred at 1 mg mL^−1^ concentration. Due to the low solubility of 4 in Tris buffer a mixture of methanol and Tris buffer (1 mg mL^−1^ of 4 in 35% MeOH/0.02 M Tris–HCl pH 7.5) was used to elute bound protein. Eluting the column with methanol in Tris buffer does not elute protein. The column is again equilibrated with Tris buffer to be used for another sample. These column can be regenerated for additional elutions (>30 runs) with 2 μL loading. The column was stable over long period of time, when stored at 4 °C.

### 3.3. Apo A1 yield

The efficiency of peptide affinity in specifically concentrating serum Apo A1 was estimated after competitive elution. The initial serum sample used for the affinity purification was 2 μL, which, based on literature concentrations of Apo A1 in chicken serum should contain ∼100 mg% (1 mg mL^−1^). Therefore, approximately 2 μg of Apo A1 was added to the column.^45^ The protein concentration of purified sample enriched in Apo A1 was determined, by Bradford assay, to be 1 μg. The yield of Apo A1 in this assay is ∼50%. The advantage of this peptide affinity method is that small volumes of serum can be used and the method can afford screening of various biological fluid samples.

### 3.4. SDS-PAGE

**Fig. 5:**
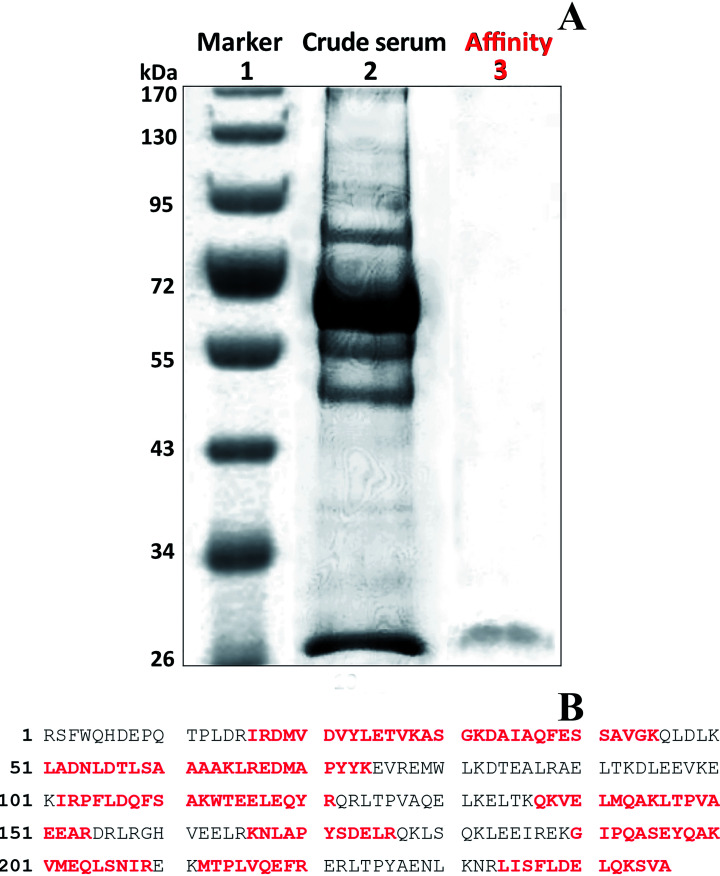
Apo A1 purification: SDS-PAGE (A) lanes 2 and 3 were loaded with 10 μg and 1 μg, respectively; Apo A1 peptide sequence in red identified by MS of tryptic fragments: total peptide sequences of the NCBI-matched protein for chicken protein (lane 3) (B).

Bound protein that was eluted with 4 was purified using SDS-PAGE electrophoresis (Fig. 5A). Coomassie blue stained a single faint band with a molecular mass of 28 kDa in the fraction. The gel containing the band was excised and the protein digested in the gel with trypsin as described in Section 2.7. The resulting peptide masses were determined and tryptic fragment masses were submitted to Mascot (Matrix Science Ltd., London, UK) for peptide search against the NCBInr database hosted by National Research Council of Canada (NRC, Ottawa, ON, Canada). The amino acid sequence was a consistent match with chicken protein and was identical to that of a chicken Apo A1 (Fig. 5B). Trypsin fragments were identified that corresponded to 58% of the Apo A1 protein.

Specific binding of chicken Apo A1 occurs with 4. The main function of Apo A1 is in transport of cholesterol and this protein affects cholesterol efflux. It was reported that CsA inversely affects the Apo A1 uptake, degradation and secretion. If LOs bind with Apo A1, then they might act as inhibitors and may interfere in cholesterol homeostasis. This study might help to elucidate the mechanism behind LO biological activity.

## 4. Conclusions

LOs were linked with solid matrix through an amide linkage with high coupling efficiency. The incorporation of LOs to the affinity media was determined by elemental analysis. This study presents the development of affinity media based on cyclic peptide for recovering proteins from mixed protein fractions using chicken serum as a model fluid. The bound LO analog had reversible affinity for a single serum protein. Tryptic digestion of the protein and analysis of the fragments revealed that the protein was Apo A1. This was consistent with the protein molecular mass of 28 kDa and trypsin digest fragment masses. It provides a quicker and less expensive alternative to chromatography-based methods for high throughput screening of Apo A1 from small amounts of serum. This is a model study and the methods will be used to study other biological fluids. Our studies suggest that the Apo A1 purified by affinity chromatography represents a specific binding protein for LO in chicken serum. This finding might also help to understand the mechanism of action of these bioactive peptides with Apo A1 and might provide an insight of their biological action.

## Conflicts of interest

There are no conflicts of interest to declare.

## Supplementary Material
