# Correction: Affinity binding of chicken apolipoprotein A1 to a novel flax orbitide (linusorb)

**DOI:** 10.1039/d0ra90034f

**Published:** 2020-04-14

**Authors:** Pramodkumar D. Jadhav, Youn Young Shim, Martin J. T. Reaney

**Affiliations:** Department of Plant Sciences, University of Saskatchewan Saskatoon SK S7N 5A8 Canada younyoung.shim@usask.ca martin.reaney@usask.ca +1 306 966 5015; Guangdong Saskatchewan Oilseed Joint Laboratory, Department of Food Science and Engineering, Jinan University Guangzhou Guangdong 510632 China; Prairie Tide Chemicals Inc. Saskatoon SK S7J 0R1 Canada

## Abstract

Correction for ‘Affinity binding of chicken apolipoprotein A1 to a novel flax orbitide (linusorb)’ by Pramodkumar D. Jadhav, *et al.*, *RSC Adv.*, 2018, **8**, 17702–17709.

The authors regret the omission of the following conflict of interest statement.

Dr Martin J. T. Reaney is the founder of, and has an equity interest in, Prairie Tide Diversified Inc. (PTD, Saskatoon, SK, Canada: previous company name is Prairie Tide Chemicals Inc.). Dr Youn Young Shim is a Market Consultant for PTD in Korea. The terms of this arrangement have been reviewed and approved by the University of Saskatchewan in accordance with its conflict of interest policies.

The Royal Society of Chemistry apologises for these errors and any consequent inconvenience to authors and readers.

## Supplementary Material

